# The involvement of protein kinase C-ε in isoflurane induced preconditioning of human embryonic stem cell - derived Nkx2.5^+^ cardiac progenitor cells

**DOI:** 10.1186/s12871-016-0178-1

**Published:** 2016-02-20

**Authors:** In-Ae Song, Ah-Young Oh, Jin-Hee Kim, Young-Min Choi, Young-Tae Jeon, Jung-Hee Ryu, Jung-Won Hwang

**Affiliations:** 1 Department of Anesthesiology and Pain Medicine, Seoul National University Bundang Hospital, Seongnam-si, Republic of Korea; 2 Department of Anesthesiology and Pain Medicine, Seoul National University College of Medicine, Seoul, Repulic of Korea; 3 Department of Obstetrics and Gynecology, Seoul National University College of Medicine, Seoul, Republic of Korea; 4 The Institute of Reproductive Medicine and Population, Medical Research Center, Seoul National University College of Medicine, Seoul, Republic of Korea

**Keywords:** Human embryonic cell, Heart failure, Preconditioning

## Abstract

**Background:**

Anesthetic preconditioning can improve survival of cardiac progenitor cells exposed to oxidative stress. We investigated the role of protein kinase C and isoform protein kinase C-ε in isoflurane-induced preconditioning of cardiac progenitor cells exposed to oxidative stress.

**Methods:**

Cardiac progenitor cells were obtained from undifferentiated human embryonic stem cells. Immunostaining with anti-Nkx2.5 was used to confirm the differentiated cardiac progenitor cells. Oxidative stress was induced by H_2_O_2_ and FeSO_4_. For anesthetic preconditioning, cardiac progenitor cells were exposed to 0.25, 0.5, and 1.0 mM of isoflurane. PMA and chelerythrine were used for protein kinase C activation and inhibition, while εψRACK and εV1-2 were used for protein kinase C -ε activation and inhibition, respectively.

**Results:**

Isoflurane-preconditioning decreased the death rate of Cardiac progenitor cells exposed to oxidative stress (death rates isoflurane 0.5 mM 12.7 ± 9.3 %, 1.0 mM 12.0 ± 7.7 % vs. control 31.4 ± 10.2 %). Inhibitors of both protein kinase C and protein kinase C -ε abolished the preconditioning effect of isoflurane 0.5 mM (death rates 27.6 ± 13.5 % and 25.9 ± 8.7 % respectively), and activators of both protein kinase C and protein kinase C - ε had protective effects from oxidative stress (death rates 16.0 ± 3.2 % and 10.6 ± 3.8 % respectively).

**Conclusions:**

Both PKC and PKC-ε are involved in isoflurane-induced preconditioning of human embryonic stem cells -derived Nkx2.5^+^ Cardiac progenitor cells under oxidative stress.

## Background

The heart is one of the least regenerative organs and irreversible damage to a large amount of cardiomyocytes may result in heart failure. There have been numerous works on cardiac stem cell therapy for the regeneration of damaged myocardium, however the results have been controversial [1, 2]. Various types of stem cells originating from both embryonic and adult human tissue sources were used for cardiac cell regeneration therapy. For example, stem cell populations such as human embryonic stem cells (hESCs), cardiac progenitor cells (CPCs), bone marrow-derived stem cells, tissue specific stem cells, and induced pluripotent stem cells have been studied for cardiac repair [1]. Each cell type had its own strengths and weaknesses and no single cell type has been proven to meet the criteria for clinical applications. CPCs hold promise for cardiac regeneration, and are known to be multipotent and proliferative, and able to differentiate into cardiomyocytes and vascular cells [3]. However, improving the graft survival in a hostile, diseased environment such as ischemic myocardium is an important issue to be resolved. For boosting the survival rates of implanted CPCs to the infarcted heart, prosurvival cocktails were used, which had a mechanism similar to anesthetic-induced preconditioning (APC) [4].

In APC, volatile anesthetics are known to have a protective effect in ischemia-reperfusion injury of myocardium. By exposing the cells to volatile anesthetics before transplantation, we expect the cells to have a greater survival rate when transplanted into a poor ischemic environment. In another study, we have shown that isoflurane has a preconditioning effect in hESCs-derived CPCs, when applied before exposure to oxidative stress [5].

Research has revealed that mitochondria, reactive oxygen species, K_ATP_ channels, and protein kinase C (PKC) play a major role in the mechanism of APC [6]. However, most of the studies on the mechanism of APC have been with animals and data from human cells are scarce. Additionally the determination of the exact isoform of PKC which has the main role in APC was controversial. In this study, we investigated the involvement of PKC and more specifically, of isoform epsilon PKC (PKC-ε) in isoflurane induced preconditioning of hESCs-derived CPCs exposed to oxidative stress because PKC-ε was shown to have a constant important role related to ischemia- or anesthesia-induced preconditioning of animal experiments [2, 7–9].

## Methods

### hESCs culture, generation of hESCs-derived CPCs and characterization of differentiated CPCs

The methods for hESCs culture, the generation of hESCs-derived CPCs and characterization of differentiated CPCs were the same as those of previous studies [5, 10, 11]. This study did not include personal information because human embryonic stem cells were obtained from the National Stem Cell Bank. Institutional review board of Institute of Reproductive Medicine and Population approval was obtained (number: IRMP-IRB2009112) and individual patient consent was waived as there were no specific human participants.

In brief, H1 hESC (WA01) was cultured on a feeder cell layer of mouse embryonic fibroblasts inactivated with mitomycin C (Invitrogen, Carlsbad, CA, USA). Colonies of hESCs were passaged every week by a mechanical micro-dissection method. After 60 % of the plates were covered with undifferentiated cell colonies for over 7 days, the cells were made into small clumps with incubation in the collagenase solution. They were then moved to suspension culture in a 24-well non-treated dish with a differentiation medium and were soaked for 4 days until the cells became embryoid bodies (EBs). A total of 20 ng/ml of bone morphogenetic protein 4 (R&D Systems, Minneapolis, MN, USA) was mixed with the differentiation medium for the suspension culture. The EBs were moved into Matrigel-coated 35-mm plates (BD Biosciences, San Jose, CA, USA) and expanded for 2–3 weeks. Immunostaining with antibodies directed against human Nkx2.5 (mouse monoclonal, R&D Systems) was done to detect differentiated CPCs. 4, 6-diamidino-2-phenylindole, DAPI (Vector Laboratories, Burlingame, CA, USA) was used to stain all nuclei. DAPI-positive cells were counted as the total number of cells and the number of Nkx2.5-positive cells was expressed as a percentage of the total number of cells. The images from 10 random microscopic fields of each plate at × 200 magnification were obtained.

### Cell survival study

**Fig. 1:**
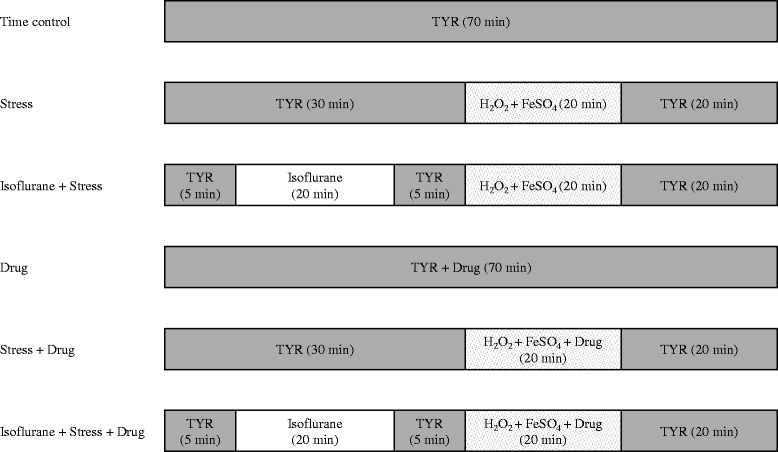
Schematic illustration of the study protocol. TYR = perfusion with Tyrode solution; Drug = chelerythrine or cell-permeable ePKC V1-2 peptide (εV1-2) or 4β-phorbol 12-myristate 13-acetate (PMA) or pseudo-receptors for activated C-kinase epsilon (εψRACK)

A cell survival study was performed using a previously reported protocol [5, 12]. The differences were the drugs for the experiments. The protocols for the experiments are illustrated in Fig. 1. In brief, differentiated CPC clusters were separated with 0.25 % of trypsin-ethylenediaminetetraacetic acid solution for 5 min and were washed in the differentiation medium. Several drops of suspension including CPCs were placed in a chamber on the stage of an inverted microscope (ECLIPSE TS100; Nikon, Tokyo, Japan) until cells were settled. We stained those with 0.5 ml of 0.4 % trypan blue solution (Sigma-Aldrich, St. Louis, MO, USA) for 2 min followed by washout with glucose-free Tyrode solution (132 mM NaCl, 10 mM, HEPES, 5 mM KCl, 1 mM CaCl_2_ and 1.2 mM MgCl_2_ adjusted to pH 7.4). Round cells that excluded trypan blue were considered living. We checked the location of living cells by a chamber bottom with a labelled grid. Approximately one hundred living CPCs were counted for 10 min in each experiment. For example, N=7 refers to the counting the numbers of about 700 living CPCs. After the cell count, the plate was washed with glucose-free Tyrode for 30 min, then the CPCs were exposed to 200 mM H_2_O_2_ and 100 mM FeSO_4_ · 7H_2_O (Sigma-Aldrich) for 20 min to simulate the oxidative stress. The CPCs were washed out with glucose-free Tyrode solution for 20 min. Then the remaining living CPCs were counted after staining with trypan blue. For APC, the cells were exposed to isoflurane dissolved Tyrode solution for 20 min before exposure to the oxidative stress. We changed all materials with a potential to contact CPCs such as plastic tubing, plastic syringes, cover glasses and slide glasses to new ones in every experiment to prevent contamination between treatment groups. Moreover we randomized the order of experiments daily and the researchers who counted the cell deaths with a microscope were blinded regarding drug treatment.

### Drugs

Isoflurane was prepared by the same method as described in the previous study [5]. Briefly, it was dissolved in glucose-free Tyrode solution by sonication and delivered to CPCs by airtight syringes. We exposed CPCs to 0.25 mM, 0.5 mM and 1.0 mM isoflurane. To confirm the concentrations of isoflurane which varied ± 10 % of the reported value, analyses were performed by gas chromatography (Shimadzu, Kyoto, Japan).

A PKC-ε inhibitor, cell permeable PKC-ε V1-2 peptide (εV1-2) and a selective PKC-ε activator, pseudo-receptors for activated C-kinase epsilon (εψRACK) were kindly provided gratis by the laboratory of Daria Mochly-Rosen (Department of Chemical and Systems Biology, Stanford University, CA). We dissolved an isoform-nonspecific PKC inhibitor, chelerythrine (1 μM; Sigma-Aldrich), and εV1-2 (1 μM) in the superfusing solution during oxidative stress to examine the effects of the PKC and PKC-ε on cell survival [13]. An isoform-nonspecific PKC activator, 4β-phorbol 12-myristate 13-acetate (PMA, 10 nM) and a selective PKC-ε activator, pseudo-receptors for activated C-kinase epsilon (εψRACK, 2 μM) were mixed with the superfusion solution for oxidative stress to illuminate the effects of the PKC and their isoform on cell survival. PMA was dissolved in dimethyl sulfoxide and εψRACK was dissolved in deionized distilled water. All stock solutions were diluted to the required concentrations in the superfusing buffer immediately before usage.

### Statistical analysis

N is the number of hESC-derived CPCs plates. Experimental groups consisted of hESC-derived CPCs from at least five separate differentiations. Predictive Analytics Software statistics version 18.0 (SPSS Inc., Chicago, IL, USA) was used for the statistical analyses. Data were analyzed using one-way analysis of variance with the Scheffé post hoc test. Differences were considered to be statistically significant if *P-* values were less than 0.05. Results are presented as means ± standard deviation.

## Results

### Differentiation and characterization of hESC-derived CPCs

**Fig. 2:**
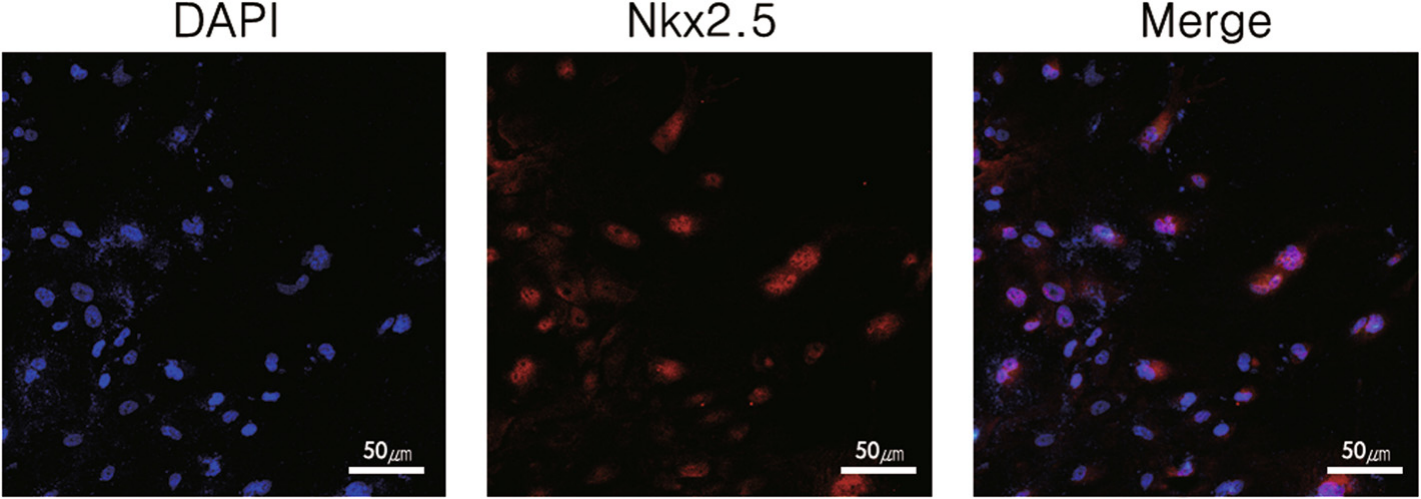
Immunostaining with DAPI, and anti-Nkx2.5 was performed to confirm cardiac differentiation of human embryonic stem cells. The merge was the merged image of DAPI and anti-Nkx2.5. Original magnification × 200. Scale bar, 50μm

Confocal microscopic examinations were done with five plates to identify CPCs derived from hESCs with early cardiac marker (Nkx2.5). The proportion of the cells stained with Nkx2.5 was 95 ± 3 % of the total cell number (Fig. 2).

### Isoflurane-induced preconditioning on hESC-derived CPCs under oxidative stress

**Fig. 3:**
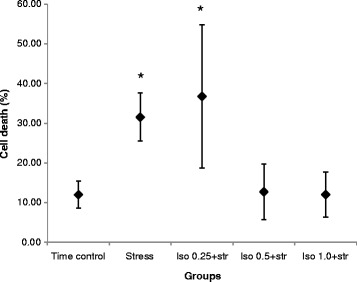
Effect of isoflurane-induced preconditioning on death rate of hESCs-derived Nkx2.5^+^ CPCs under oxidative stress. Preconditioning with 0.25 mM of isoflurane could not lower the death rate of CPCs under oxidative stress. However, preconditioning with 0.5 mM and 1.0 mM of isoflurane decreased death rate of CPCs. * Statistically significant differences with group time control, group Iso 0.5 + str, and group Iso 1.0 + str (*P* < 0.05). Each black bar represents the 95 % confidence interval and a black diamond the mean value of the death rate of CPCs. Iso 0.25 + Str = 0.25 mM of isoflurane plus stress; 0.5 Iso + Str = 0.5 mM of isoflurane plus stress; 1.0 Iso + Str = 1.0 mM of isoflurane plus stress

Glucose-free Tyrode solution did not have an effect on the death rate of CPCs in the time-control group (9.6 ± 5.4 %, *n* = 7). Oxidative stress increased the CPCs death rate to 31.4 ± 10.2 % (*n* = 11). For the evaluation of the preconditioning effect of isoflurane, three concentrations were used. Preconditioning with 0.25 mM of isoflurane did not lower the death rate of CPCs (36.7 ± 18.0 %, *n* = 10). However, 0.5 mM and 1.0 mM of isoflurane decreased CPCs death rate to 12.7 ± 9.3 % (*n* = 7) and 12.0 ± 7.7 % (*n* = 7) respectively without a significant difference between the two concentrations (Fig. 3).

### The involvement of PKC in isoflurane-induced preconditioning on hESC-derived CPCs under oxidative stress

**Fig. 4:**
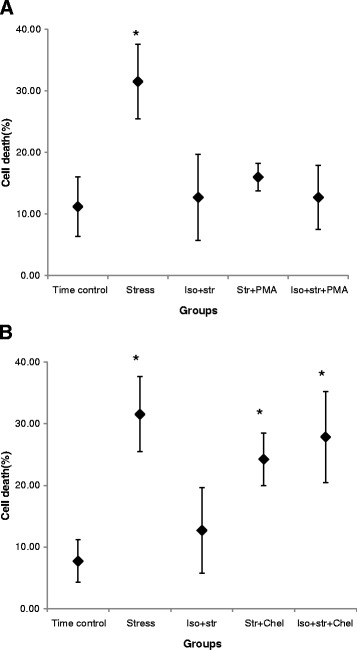
The role of PKC on 0.5 mM of isoflurane-induced preconditioning of hESCs-derived Nkx2.5^+^ CPCs under oxidative stress. **a** 4β-phorbol 12-myristate 13-acetate (PMA), an isoform-nonspecific PKC activator, induced the effect of preconditioning. * Statistically significant differences with group time control, group Iso + str, group str + PMA, and group Iso + str + PMA (*P* < 0.05). **b** Chelerythrine, an isoform-nonspecific PKC inhibitor, abolished the preconditioning effect of isoflurane. * Statistically significant differences with group time control, and group Iso + str (*P* < 0.05). Each error bar represents the 95 % confidence interval and a diamond indicates the mean value of the death rate of CPCs. Iso + Str = isoflurane plus stress; Str + Chel = stress plus chelerythrine; Iso + Str + Chel = isoflurane plus stress Chelerythrine; Str + PMA = stress plus PMA; Iso + Str + PMA = isoflurane plus stress PMA

PMA and chelerythrine themselves had no effect on the death rate of CPCs in the time-control groups (11.7 ± 5.9 %, *n* = 7 and, 8.9 ± 3.8 %, *n* = 7). PMA had a protective effect on CPCs when they were under oxidative stress: the death rates of CPCs were 16.0 ± 3.2 % (*n* = 8). When CPCs were treated with PMA and 0.5 mM of isoflurane, PMA did not reduce or potentiate the protective effect of isoflurane (12.7 ± 7.0 %, *n* = 7) (Fig. 4a). Chelerythrine did not show additional effects on the death rate of CPCs under oxidative stress (24.1 ± 6.1 %, *n* = 8). However, it abolished the preconditioning effects of isoflurane on CPCs under oxidative stress (27.6 ± 13.5 %, *n* = 13) (Fig. 4b).

### The involvement of PKC-ε in isoflurane-induced preconditioning on hESC-derived CPCs under oxidative stress

**Fig. 5:**
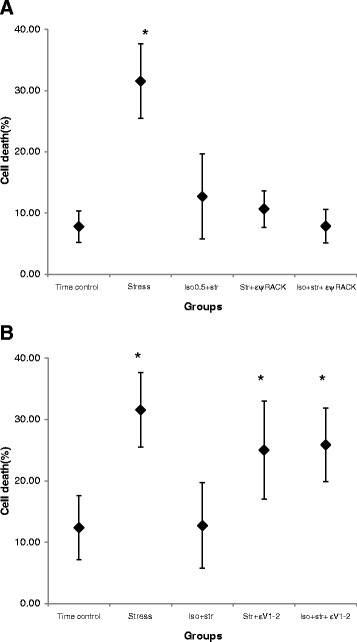
The role of PKC-ε on 0.5 mM of isoflurane-induced preconditioning of hESCs-derived Nkx2.5^+^ CPCs under oxidative stress. **a** εψRACK, a selective PKC-ε activator, induced the effect of preconditioning. * Statistically significant differences with group time control, group Iso + str, group str + εψRACK, and group Iso + str + εψRACK (*P* < 0.05). **b** a cell-permeable epsilon PKC V1-2 peptide (εV1-2), a selective PKC-ε inhibitor, abolished the preconditioning effect of isoflurane. * Statistically significant differences with group time control, and group Iso + str (*P* < 0.05). Each error bar shows the 95 % confidence interval and a diamond denotes the mean value of the death rate of CPCs. Iso + Str = isoflurane plus stress; Str + εV1-2 = stress plus εV1-2; Iso + Str + εV1-2 = isoflurane plus stress εV1-2; Str + εψRACK = stress plus εψRACK; Iso + Str + εψRACK = isoflurane plus stress εψRACK

There were no significant effects on the death rate of CPCs by εψRACK and εV1-2 themselves in the time-control group (7.8 ± 2.4 %, *n* = 8, and 12.8 ± 5.7 %, *n* = 8). εψRACK had a protective effect on CPCs when they were under oxidative stress: the death rates of CPCs were 10.6 ± 3.6 % (*n* = 8). When εψRACK was added after isoflurane wash out, it did not reduce or potentiate the protective effect of 0.5 mM of isoflurane (7.8 ± 3.7 %, *n* = 7) (Fig. 5a). εV1-2 did not show additional effects on the death rate of CPCs when they were under oxidative stress (25.2 ± 8.0 %, *n* = 8). However, it abolished the preconditioning effect of isoflurane on CPCs under oxidative stress (25.9 ± 8.7 %, *n* = 8) (Fig. 5b).

## Discussion

This is, to our knowledge, the first study showing the involvement of PKC and PKC-ε in APC using human cells. Our data showed that PKC, and specifically PKC-ε involved in isoflurane-induced preconditioning of hESCs-derived Nkx2.5^+^ CPCs under oxidative stress because the inhibitors of PKC and PKC-ε both abolished the preconditioning effect of isoflurane and the activators of both PKC and PKC-ε showed the protection of hESCs-derived Nkx2.5^+^ CPCs from oxidative stress.

PKC seemed to play an important role in ischemic preconditioning (IPC), with cardioprotection effect after short ischemia-reperfusion injury. However PKCs have at least 12 isoforms and the exact roles of each isoform in ischemic preconditioning are not fully elucidated and could well differ in studies using different animal species and materials [14]. For example, PKC-ε and –δ in rats, PKC-α in a canine model, PKC-α and –ε in adult human right atrial appendage harvested from open heart surgeries, are known to play an essential role in IPC [14–17]. Certain isoforms, like PKC-δ, are known to act contrarily in ischemic preconditioning. That is, inhibiting PKC-δ showed cardioprotection from simulated ischemia in rat and mouse [18].

APC seems to be a novel method of cardioprotection from ischemic injury which could be done safely without risk of myocardial ischemia, which was a weak point of IPC. It may be equally as effective as IPC and have some mechanisms in common with IPC, as we showed both previously and in this study with isoflurane-induced APC [5, 6].

Although the mechanism of APC has been studied, it is still not clear enough. The keynote of the mechanism of APC might be in the mitochondria as the end effecters of ischemia and reperfusion injury and as the determinant of cell death. That is, the opening of mitochondrial permeability transition pores permit cellular Ca^2+^ overload and subsequent cell death [19]. Several studies implied APC might delay the opening of mitochondrial permeability transition pores via PKC-ε mediated inhibition in rat myocardium [20]. Current study also implied that PKC- ε also involved in the APC mechanism of humans.

We used hESCs which could differentiate into various cells, including cardiomyocytes, and can play an important role in pathophysiologic or pharmacologic studies on cardiac disease. Indeed, these hESC-derived cardiomyocytes make action potential, utilize calcium rendering it transient and show excitation-contraction coupling [21, 22].

The hESC-derived cardiomyocytes were transplanted to treat infarcted rat heart but the results are controversial [4, 23]. The efficacy of transplanted hESC-derived cardiomyocytes depended on the ability to survive in the poor environment of the host. CPCs could also be used for transplantation and show a higher survival rate after transplantation than cardiomyocytes. The microenvironment of transplanted cells could provide cues for further differentiation into mature cells such as vascular smooth muscle cells, endothelial cells or cardiomyocytes [24]. There were clinical trials using CPCs after myocardial infarction showing improvement of myocardial function or the reduction of the scar mass with increase of regional contractility and with no adverse effect until 6 months or 1 year [25, 26]. Our results might contribute to a method of improving the survival of CPCs engrafted to a hostile environment thus increasing the success rate of cardiac regeneration therapy.

### Limitations

Our data showed the possibilities of using the activator of PKC or PKC-ε instead of preconditioning by artificially inducing ischemia or by exposure to anesthetics for the prevention of ischemic injury, making the procedure more convenient by simple injection of drugs. However, the study hinges on the specificity of the PKC inhibitors and activators, and is largely pharmacologic in nature. The lack of control peptides is a limitation. The safety of the drugs should be elucidated further before clinical application. For example, PMA, a non-specific PKC activator is known to be a carcinogen [27]. In addition, nonspecific activators of PKC should be used with caution, although our data showed protective effects, because some of isoforms are known to have the opposite effects [18]. Accordingly PKC-ε activator like εψRACK might be a promising choice in this aspect, however further evaluation concerning efficacy and toxicity in human beings is also required. Another limitation was whether the study with hESCs could reflect the situation of the real human hearts in vivo as this study was exclusively performed in tissue culture experiments, which is required for future studies in the clinical setting.

## Conclusions

Both PKC and PKC-ε might be involved in isoflurane-induced preconditioning of hESCs-derived Nkx2.5^+^ CPCs under oxidative stress.

## Abbreviations

APC: anesthetic-induced preconditioning
Ca^2+^: ionized Calcium
CPCs: cardiac progenitor cells
DAPI: 4, 6-diamidino-2-phenylindole
EBs: embryoid bodies
hESCs: human embryonic stem cells
IPC: ischemic preconditioning
PKC: protein kinase C
PKC-ε: isoform epsilon PKC
PMA: An isoform-nonspecific PKC activator, 4β-phorbol 12-myristate 13-acetate
εV1-2: An PKC-ε inhibitor, cell permeable PKC-ε V1-2 peptide
εψRACK: a selective PKC-ε activator, pseudo-receptors for activated C-kinase epsilon
